# The pseudogene-derived long non-coding RNA SFTA1P suppresses cell proliferation, migration, and invasion in gastric cancer

**DOI:** 10.1042/BSR20171193

**Published:** 2018-04-20

**Authors:** Hongwei Ma, Tianshi Ma, Miao Chen, Zigui Zou, Zhihong Zhang

**Affiliations:** 1 Department of Pathology, The First Affiliated Hospital of Nanjing Medical University, Nanjing, Jiangsu, People’s Republic of China; 2 Department of Pathology, Affiliated People’ Hospital of Jiangsu University, Zhenjiang, Jiangsu, People’s Republic of China

**Keywords:** gastric cancer, lncRNA, pseudogene, SFTA1P

## Abstract

Pseudogenes were once regarded as transcriptionally inactive and without specific molecular function. However, current evidence shows that pseudogene-derived long non-coding RNAs (lncRNAs) may be crucial regulators of human cancer development, including gastric cancer (GC). In the present study, we report that a pseudogene-derived lncRNA named surfactant associated 1, pseudogene (SFTA1P), which is 693-nt long, was significantly down-regulated in GC tissues compared with that in the adjacent normal tissues. In addition, decreased SFTA1P expression was strongly correlated with advanced tumor lymph node metastasis (TNM) stage, larger tumor size, lymphatic metastasis, and poor prognosis of patients with GC. Moreover, gain-of-function experiments revealed that the overexpression of SFTA1P inhibits cell proliferation, migration, and invasion, thus verifying the tumor inhibitory role of SFTA1P in GC. Furthermore, we investigated the potential action mechanism of SFTA1P. Our results showed that down-regulation of SFTA1P may be associated with decreased TP53 expression. In summary, our work suggests that the pseudogene-derived lncRNA SFTA1P functions as a tumor suppressor in GC and thus may act as a potential diagnostic and therapeutic target of GC.

## Introduction

Gastric cancer (GC) is the fifth most commonly diagnosed cancer and the third leading cause of cancer deaths globally. It is worth mentioning that its incidence rates are the highest in East Asia, especially in Japan and China [1]. Despite the comprehensive application of chemotherapy, radiotherapy, surgical treatment, and molecular-targetted treatment, the survival rate of patients with GC remains dissatisfying [2–4]. Thus, there is a pressing need to understand the mechanisms underlying GC development. Recently, many proto-oncogenes and anti-oncogenes have been verified to play crucial roles in GC oncogenesis and development. However, the specific molecular mechanisms and biomarkers involved in GC development are still poorly understood. Therefore, it is important for improving the diagnosis, treatment, and prognosis of patients with GC to further decipher its molecular mechanisms.

Whole-genome sequencing of humans led to the discovery that just 2% of human DNA encodes proteins, and the rest of it comprises non-coding segments such as pseudogenes [5–7]. However, we now know that these non-coding segments play essential roles not only in neurological, cardiovascular, developmental, and other normal processes but also in tumorigenesis [8]. Amongst these non-coding segments are genomic loci called pseudogenes. Though similar to true genes, they are deemed biologically inconsequential. This is because pseudogenes harbor early stop codons, deletions, insertions, and frameshift mutations that prevent their translation into functional proteins [9]. Although pseudogenes were classified as non-functioning genomic fossils initially in 1977 [10], pseudogene-derived RNAs can function as antisense RNAs (asRNAs), endogenous siRNAs, and competing endogenous RNAs (ceRNAs) during post-transcriptional regulation [11]. Moreover, many studies have verified that several pseudogenes participate in multiple biological processes, and their deregulation is associated with human diseases, especially cancers [12–14]. Long non-coding RNAs (lncRNAs) are defined as transcripts that are longer than 200 nts and do not encode proteins [15]. Although they were once regarded as ‘transcriptional noise’, it is now well known that lncRNAs play a key role in regulating multiple levels of gene expression during diverse biological processes [16,17]. A large number of studies have shown that lncRNAs played important roles in cancer cell proliferation, cell cycle progression, apoptosis, invasion, migration, and metastasis [18,19]. Recently, numerous studies have indicated that many pseudogene-derived lncRNAs play a crucial role in cancer progression and development. For instance, the pseudogene-derived lncRNA, FER1L4, is down-regulated in GC. FER1L4 regulates the expression of the tumor suppressor gene *PTEN* by acting as a ceRNA, thus functioning as a tumor suppressor [20]. The pseudogene-derived lncRNA SUMO1P3, is up-regulated in GC and signifies poor prognosis for patients with GC [21]. Our previous study indicated that a pseudogene-derived lncRNA DUXAP8 is up-regulated in GC. Furthermore, we found that DUXAP8 promotes cell proliferation and migration in GC by silencing PLEKHO1 expression epigenetically [22]. Hence, pseudogenes are crucial to tumorigenesis, but the overall molecular mechanisms of lncRNAs action and their expression by pseudogenes are still under investigation.

On account of the significance of pseudogenes for GC progression, we investigated the pseudogene-derived lncRNA, surfactant associated 1, pseudogene (SFTA1P), which is 693 nts long and is located on 10p14. A previous study revealed that SFTA1P is down-regulated in, and suppresses cell migration and invasion in, lung adenocarcinoma (LUAD) [23]. However, the biological function and expression pattern of SFTA1P in other tumors such as GC are still unknown. Therefore, we decided to study the function of SFTA1P in GC. We found that SFTA1P expression was down-regulated in GC tissues. Our study further indicated that SFTA1P down-regulation was associated with a poor prognosis for patients with GC. Additionally, gain-of-function assays revealed that SFTA1P can inhibit GC cell proliferation, as well as restrain cell migration and invasion. It is well known that TP53 acts as a tumor suppressor by inducing cell cycle arrest and apoptosis [24]. Our study suggests that TP53 might mediate the effect of SFTA1P on cell proliferation, migration, and invasion. Taken together, our work shows that SFTA1P could serve as a tumor suppressor and may serve as a marker for GC diagnosis or as a biological target for treating GC.

## Materials and methods

### Tissue samples and clinical feature collection

We collected the 68 pairs of GC tissues and adjacent normal tissues from primary GC patients at the First Affiliated Hospital of Nanjing Medical University (Nanjing, Jiangsu, China). No radiotherapy and chemotherapy was provided to the patients, before the surgery. Patients were diagnosed with GC according to histopathological evaluation and their clinicopathological characteristics are shown in Table 1. The 68 pairs tissue samples were immediately stored at −80°C. This experiment was allowed by the Research Ethics Committee of Nanjing Medical University. We received all the informed consents.

**Table 1:** Correlation between SFTA1P expression and clinicopathological characteristics of GC patients

Characteristics	*n* (%)	SFTA1P		*P*
		Low NO. cases (34)	High NO. cases (34)	Chi-squared test *P*-value
**Gender**				0.806
Male	39 (57.4%)	19	20	
Female	29 (42.6%)	15	14	
**Age**				0.329
≤65	30 (44.1%)	13	17	
≥65	38 (55.9%)	21	17	
**Stage**				0.002*
I + II	33 (48.5%)	10	23	
III	35 (51.5%)	24	11	
**Lymph node metastasis**				0.029*
Negative	33 (48.5%)	12	21	
Positive	35 (51.5%)	22	13	
**Tumor size**				0.026*
≤5 cm	27 (39.7%)	9	18	
>5 cm	41 (60.3%)	25	16	

* *P*-value<0.05 was considered significant.

### Cell culture

We used BGC823 and SGC7901, two GC cell lines, and GES-1, the normal gastric epithelial cell line (Shanghai, China). RPMI 1640 medium (GIBCO-BRL) was used for BGC823 cells, however, SGC7901 cells need to be cultured in DMEM (GIBCO-BRL) medium. The medium was added with 10% FBS and two antibiotics (100 mg/ml streptomycin and 100 U/ml penicillin). The cells were held in wet air atmosphere at 37°C with 5% CO_2_.

### RNA extraction and qRT-PCR analyses

We used TRIzol reagent (Invitrogen) to extract RNA from tissues and all cultured cells. Then, we reverse transcribed RNA to cDNA (Takara, Dalian, China). Real-time PCR analysis was performed to test the expression of genes by using SYBR Premix Ex Taq (Takara). Results were normalized to the expression of glyceraldehyde-3-phosphate dehydrogenase (GAPDH). The specific primer sequences were:

SFTA1P F, CAGCATTCCAGGTGGGCTTT,
SFTA1P R, CCTTGTTTGGCTTACTCGTGC;
GAPDH F, GGGAGCCAAAAGGGTCAT,
GAPDH R, GAGTCCTTCCACGATACCAA.

### Transfection of GC cells

We generated the pCDNA-SFTA1P vector (Genechem, Shanghai, China) for overexpression SFTA1P in GC cells. The empty vector was used as a control. We transfected the GC cells with pCDNA-SFTA1P vector or empty vector by using Lipofectamine 2000 (Invitrogen, U.S.A.), then the cells were incubated for 48 h. After 48 h of transfection, GC cells were collected for a series of assays.

### Cell proliferation assays

Cell proliferation was detected by using MTT assays (Roche Applied Science). GC cells which transfected with pCDNA-SFTA1P or empty vector were seeded in 96-well plates. The cell viability was monitored every 24 h. Colony formation analysis: the transfected cells were maintained in 10% FBS for 14 days in the six-well plates, the medium was changed every 3 days. At last, the colonies were disposed with methanol, colored with Crystal Violet (Sigma–Aldrich). Then, we counted the visible colonies and the experiments were assessed in triplicate independently.

### EdU analysis

We performed EdU analysis (Ribobio, Guangzhou,China) to test the cell proliferation. GC cells which transfected with pCDNA-SFTA1P or empty vector were put in 96-well plates. After 48 h transfection, we added 50 μM EdU labeling medium into the cells and incubated the cells at 37°C under 5% CO_2_ for 2 h. Then, we used paraformaldehyde for 30 min, and Triton X-100 for 20 min. After the treatment, cells were stained with anti-EdU working solution for 30 min. We used DAPI as label cell nucleus. At last, we calculated the percentage of EdU-positive cells under fluorescent microscopy.

### Flow cytometric analysis

GC cells which transfected with pCDNA-SFTA1P or empty vector were collected by trypsinization. After the double staining with FITC-Annexin V and Propidium Iodide (PI) by using the FITC Annexin V Apoptosis Detection Kit (BD Biosciences) according to the manufacturer’s recommendation, cells were analysed by flow cytometry (FACScan®; BD Biosciences). And the cell cycle analysis was stained with PI using the CycleTEST™ PLUS DNA Reagent Kit (BD Biosciences) according to the manufacturer’s recommendations, then analyzed by FACScan. The percentage of the cells in G_0_/G_1_, S, and G_2_/M phases were counted and compared.

### Cell migration and invasion assays

Transwell assays were performed to test the GC cell migration and invasion ability. In migration assays, 3×10^4^ cells were suspended in 300 µL medium containing 1% fetal bovine serum and placed into the upper chamber. In invasion assays, 1 ×10^5^ cells were placed into the upper chamber of an insert coated with Matrigel (Sigma–Aldrich, St Louis, MO, USA). The down chamber was added with corresponding medium, which contained 10% fetal bovine serum. After 24-h incubation, the cells that had migrated or invaded through the membrane were stained with methanol and 0.1% crystal violet, imaged, and counted using an IX71 inverted microscope.

### Tumor formation assay in a nude mouse model

Male BALB/c mice without thymus (5 weeks old) were under aseptic conditions, and operated upon them according to the protocols with the agreement of Shanghai Medical Experimental Animal Care Commission. SGC7901 cells were stably transfected into pCDNA-SFTA1P or empty vector. After 48 h of transfection, we collected the cells from six-well plates; 100 μl suspension cells was injected into the posterior flank of each mouse subcutaneously. We examined the tumor growth every 3 days and calculated the tumor volumes. In 15 days post-injection, the mice were killed and checked the tumor growth.

### Immunohistochemical analysis

Primary tumors were immunostained for Ki-67 as previously described [25].

### Western blot

Cell protein lysate separation of SDS/PAGE (10% gel) were transferred to 0.22 μm NC membranes (Sigma), then incubated with their antibodies. GAPDH was used as a control.

### Statistical analysis

The Student’s *t* test, χ^2^ test, or Wilcoxon test significance were conducted to analyze the differences between groups. *P*-values less than 0.05 were considered significant and all statistical analysis were used by SPSS 17.0 software (IBM, SPSS, U.S.A.).

## Results

### SFTA1P expression is down-regulated in human GC tissues

**Figure 1:**
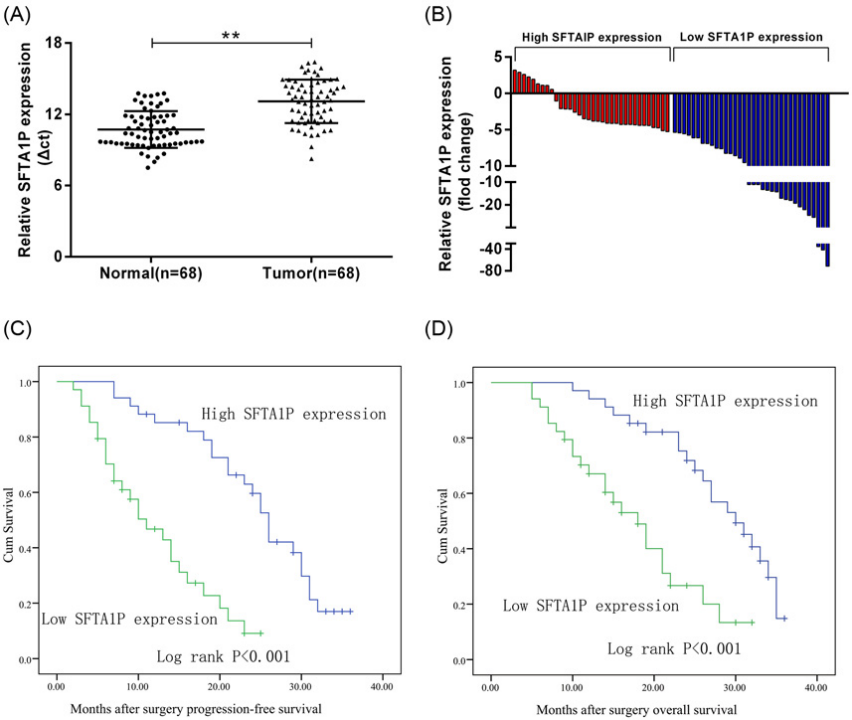
Relative SFTA1P expression in GC tissues and its clinical significance (**A**) Relative expression of SFTA1P in human GC tissues (*n*=68) compared with corresponding non-tumor tissues (*n*=68). SFTA1P expression was examined by qRT-PCR and normalized to GAPDH expression (shown as Δ*c*_t_). (**B**) Results are presented as the fold-change in tumor tissues relative to normal tissues, and SFTA1P expression was classified into two groups. (**C**,**D**) Kaplan–Meier progression-free survival and overall survival curves according to SFTA1P expression level. Error bars indicate mean ± S.E.M. *P-value <0.05, **P-value <0.01.

We performed qRT-PCR to measure SFTA1P expression in 68 pairs of human GC tissues and corresponding non-tumor normal tissue (all normalized to GAPDH expression). The results showed that the expression of SFTA1P was down-regulated in GC tissues significantly compared with its expression in corresponding non-tumor normal tissues (*P*-value <0.01, Figure 1A). In 58 cases, SFTA1P expression decreased at least two-fold in GC tissues (Figure 1B).

### Down-regulation of SFTA1P is associated with poor prognosis

In order to test whether the expression of SFTA1P was related to the clinical characteristics of patients with GC, shown in Figure 1B, the 68 patients with GC in the present study were split into two groups based on their SFTA1P expression (normalized ratio) relative to the median ratio of SFTA1P expression: high SFTA1P group (*n*=34, SFTA1P expression ratio ≥ median ratio) and low SFTA1P group (*n*=34, SFTA1P expression ratio < median ratio). The patients’ clinicopathological features are shown in Table 1. Low expression of SFTA1P showed significant correlations with advanced tumor lymph node metastasis (TNM) stage (*P*-value =0.002), lymph node metastasis (*P*-value =0.029), and larger tumor size (*P*-value =0.026). Nevertheless, SFTA1P expression did not show a significant relationship with sex (*P*-value =0.806) and age (*P*-value =0.329; Table 1).

We used the Kaplan–Meier method to examine the association between SFTA1P expression levels and the prognosis of patients with GC (Figure 1C,D). The analysis showed that patients with lower SFTA1P expression levels showed significantly shorter progression-free survival (Figure 1C, log-rank *P*-value <0.001) and overall survival (Figure 1D, log-rank *P*-value <0.001) than patients with higher expression levels of SFTA1P. These results corroborated our finding that the down-regulation of SFTA1P is associated with poor prognosis for patients with GC.

### SFTA1P inhibits GC cell proliferation *in vitro*

**Figure 2:**
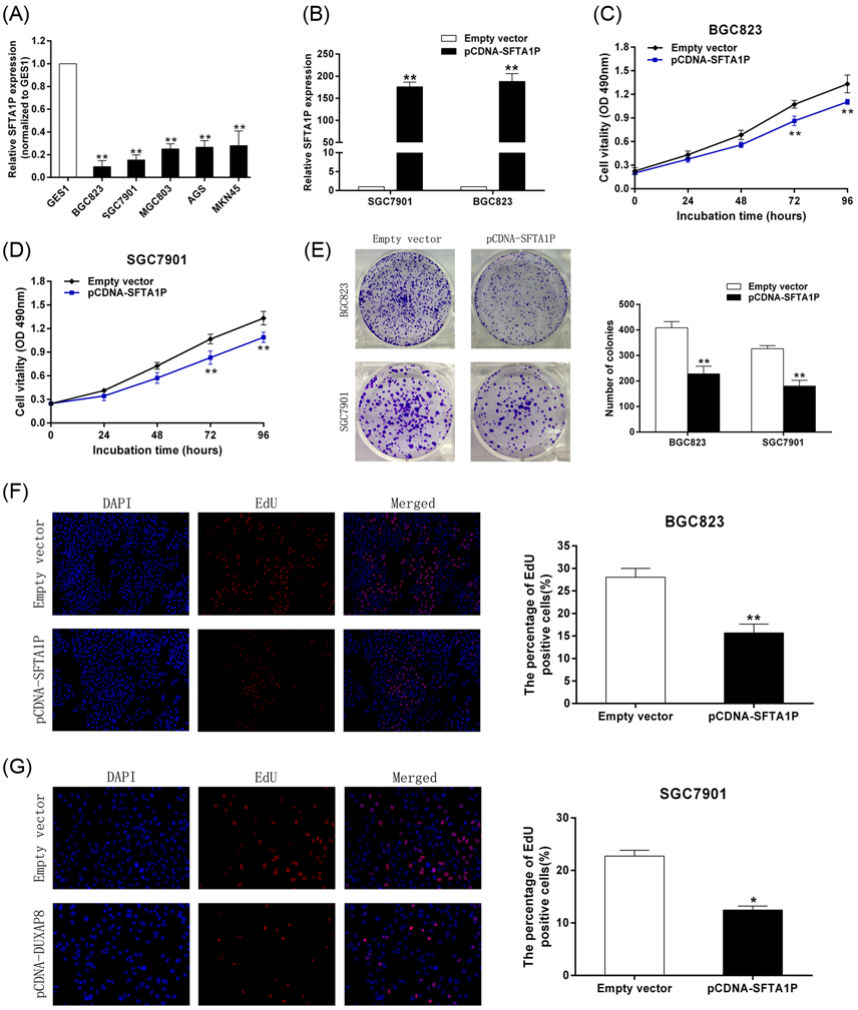
SFTA1P inhibits GC cell proliferation *in vitro* (**A**) qRT-PCR analysis of SFTA1P expression in the normal gastric epithelium cell line (GES1) and GC cells. (**B**) qRT-PCR analysis of SFTA1P expression in empty vector, pCDNA-SFTA1P transfected GC cells. (**C**,**D**) MTT assays were used to determine the viability of pCDNA-SFTA1P transfected GC cells. (**E**) Colony formation assays were performed to determine the proliferation of pCDNA-SFTA1P transfected GC cells. Colonies were counted and captured. (**F**,**G**) Proliferating BGC823 and SGC7901 cells were labeled with EdU. The Click-it reaction revealed EdU staining (red). Cell nuclei were stained with DAPI (blue). Representative images and data based on three independent experiments. Error bars indicate mean ± S.E.M. *P-value <0.05, **P-value <0.01.

In order to study the effect of SFTA1P on GC cells, qRT-PCR analysis was used to measure SFTA1P expression in some human GC cell lines. The expression of SFTA1P was noticeably down-regulated in BGC823 and SGC7901 cells (Figure 2A). Therefore, we transfected them with pCDNA-SFTA1P vector to up-regulate the expression of SFTA1P. We found that the expression of SFTA1P in pCDNA-SFTA1P-transfected GC cells significantly increased compared with its expression in the control group 48 h after transfection (Figure 2B).

Next, we performed MTT assays to test the contribution of SFTA1P to GC cell proliferation. The growth of GC cells declined significantly after SFTA1P overexpression compared with the growth of the control group (Figure 2C,D). In addition, the colony formation ability of BGC823 and SGC7901 cells decreased significantly upon overexpression of SFTA1P (Figure 2E). EdU immunostaining assays showed the same results: the number of EdU-possitive cells decreased significantly after overexpression of SFTA1P (Figure 2F,G). These results support our hypothesis that SFTA1P acts as a tumor suppressor gene and prevents GC cell proliferation.

### Overexpression of SFTA1P promotes G_1_ arrest and causes apoptosis of GC cells *in vitro*

**Figure 3:**
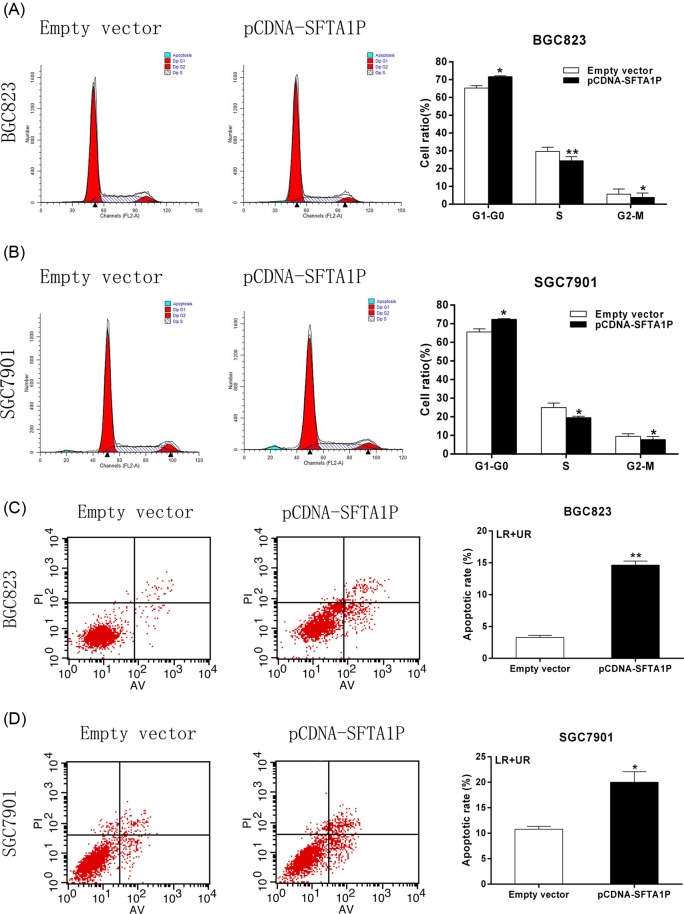
Effect of SFTA1P on GC cell apoptosis and cell cycle regulation *in vitro* (**A**,**B**) Flow cytometry was used to detect the cell cycle regulation. The bar chart represented the percentage of BGC823 and SGC7901 cells in G_0_/G_1_, S, or G_2_/M phase, as indicated. (**C**,**D**) Flow cytometry was used to detect the apoptotic rates of cells. Error bars indicate mean ± S.E.M. *P-value <0.05, **P-value <0.01. Abbreviations: LR, early apoptotic cell; UR, terminal apoptotic cell.

Two important indicators of GC cell proliferation are cell cycle stage and the level of apoptosis. Therefore, we performed flow cytometric analysis to detect the level of apoptosis and cell cycle stage of GC cells. As shown in Figure 3A,B, overexpression of SFTA1P caused a significant number of cells to undergo G_0_/G_1_-phase arrest, meanwhile, the number of S-phase cells decreased noticeably (*P*-value <0.05). The results suggest that SFTA1P is involved in cell cycle regulation. Additionally, cell apoptosis analysis showed that the number of apoptotic cells was significantly increased following SFTA1P overexpression compared with the control group (Figure 3C,D). These results reveal that SFTA1P might affect GC cell proliferation by regulating cell cycle and apoptosis.

### SFTA1P inhibits GC cell migration and invasion *in vitro*

**Figure 4:**
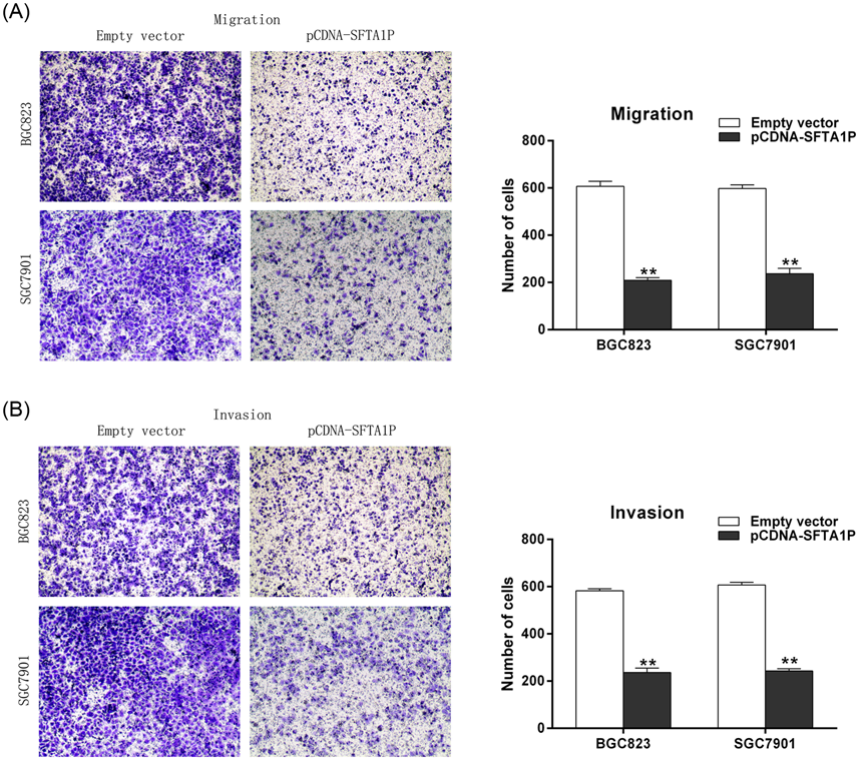
SFTA1P overexpression inhibits GC cells migration and invasion (**A**) Transwell assays were used to investigate the changes in migratory abilities of SFTA1P overexpression cells. (**B**) Transwell assays were used to investigate the changes in invasive abilities of SFTA1P overexpression cells. Error bars indicate mean ± S.E.M. *P-value <0.05, **P-value <0.01.

Cell metastasis is an important aspect of cancer progression, and may worsen the prognosis of patients with GC. Therefore, we assessed the migration and invasion abilities of GC cells by performing transwell analysis. Overexpression of SFTA1P significantly decreased the number of migratory cells (Figure 4A), thus indicating that SFTA1P can inhibit the migratory ability of GC cell. A parallel invasion assay was also performed, and the results showed that SFTA1P overexpression impeded invasion compared with invasion by control cells (Figure 4B). These results show that SFTA1P has tumor-suppressive properties that can inhibit GC cell migration and invasion.

### SFTA1P suppresses tumorigenesis of GC cells *in vivo*

**Figure 5:**
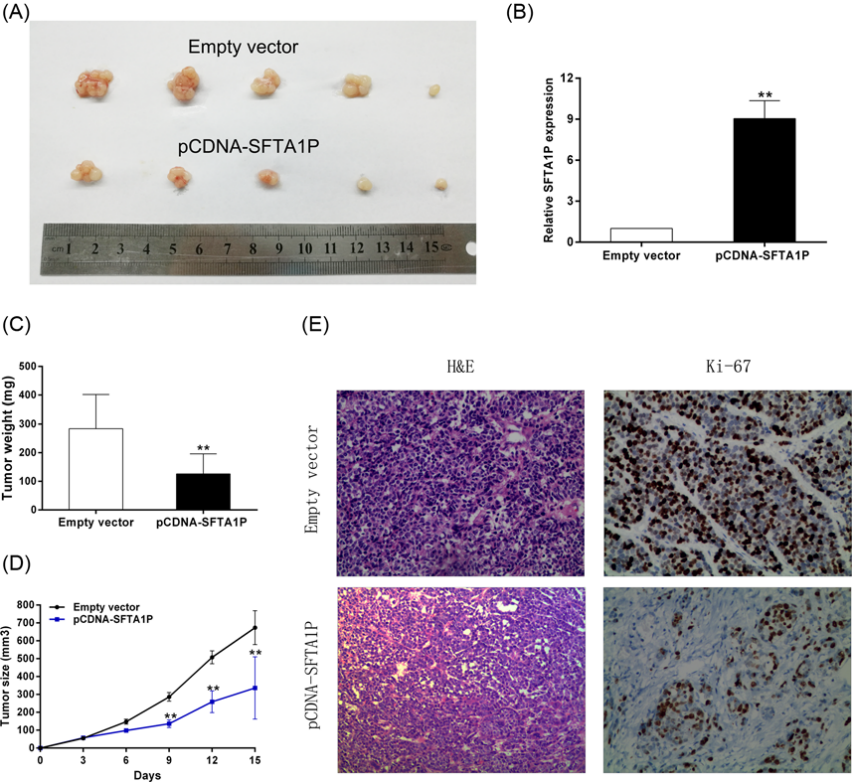
SFTA1P overexpression inhibits tumorigenesis of GC cells *in vivo* (**A**) Empty vector or pCDNA-SFTA1P were transfected into SGC7901 cells, which were injected in the nude mice (*n*=5), respectively. Tumors formed in pCDNA-SFTA1P group were dramatically smaller than the control group. (**B**) qRT-PCR was performed to detect the average expression of SFTA1P in xenograft tumors. (**C**) Tumor weights were represented as means of tumor weights ± S.D. (**D**) Tumor volumes were calculated after injection every 3 days. Points, mean (*n*=5); bars indicate S.D. (**E**) The tumor sections were under H&E staining and immunohistochemical (IHC) staining using antibodies against Ki-67. Error bars indicate mean ± S.E.M. *P-value <0.05, **P-value <0.01.

In order to test the effect of SFTA1P on tumorigenesis *in vivo*, SGC7901 cells transfected with pCDNA-SFTA1P or the empty vector were injected into nude mice. Two weeks later, the average tumors size of the pCDNA-SFTA1P-transfected group was significantly smaller than that of the control group (Figure 5A). We also measured SFTA1P expression levels in the pCDNA-SFTA1P group and the control group, and found that SFTA1P expression level of the pCDNA-SFTA1P group was much higher than that of the empty vector-transfected group (Figure 5B). Additionally, the average tumor weight of the pCDNA-SFTA1P group was significantly lower than that of the control group (Figure 5C). In addition, tumor growth in the pCDNA-SFTA1P transfected group was remarkably slower than in the empty vector-transfected group (Figure 5D). Moreover, the tumors of the pCDNA-SFTA1P transfected group displayed lower intensity of Ki-67 staining than the tumors of the empty vector-transfected group (Figure 5E). These results reveal that the down-regulation of SFTA1P is significantly correlated with GC proliferation *in vivo*.

### Potential target gene of SFTA1P in GC

**Figure 6:**
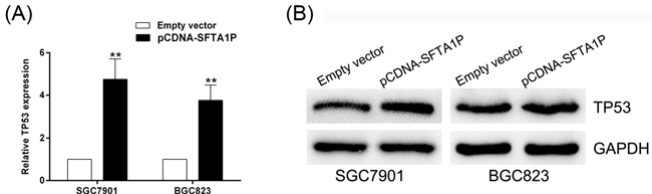
Potential targets involved in SFTA1P tumor suppressor function (**A**) qRT-PCR analysis of TP53 expression in empty vector, pCDNA-SFTA1P transfected GC cells. (**B**) The expression level of TP53 protein using Western blot analysis. Error bars indicate mean ± S.E.M. *P-value <0.05, **P-value <0.01.

To explore the potential mediators that are involved in the tumor suppressor function of SFTA1P, we tested many genes using qRT-PCR analysis. TP53, a well-known tumor suppressor, is involved in SFTA1P-associated suppression of GC. As shown in Figure 6A, TP53 expression significantly increased in the pCDNA-SFTA1P-transfected group compared with its expression in the control group. In addition, we performed Western blot analysis to measure the level of TP53 protein after overexpressing SFTA1P (Figure 6B). Our results showed that SFTA1P overexpression can promote TP53 expression both at the mRNA and protein levels in GC cells.

## Discussion

With the emergence of next-generation sequencing, the study of human genomics has experienced a great leap forward. Pseudogenes were earlier assumed to be non-functional and labeled as ‘junk DNA’, but mounting evidence has highlighted the importance of pseudogenes in human diseases including cancers [26]. At the same time, increasing evidence suggests that alterations in the expression of different lncRNAs is one of the drivers of tumorigenesis [16,17]. LncRNA is an RNA transcript that is 200-nt long with no capacity for encoding proteins [27]. However, increasing studies have shown that the dysregulation of pseudogene-derived lncRNAs contributes to the pathogenesis of GC [20–22]. Thus, the biological mechanisms by which pseudogene-derived lncRNAs operate needs intensive investigations to provide lncRNA-based biomarkers against diseases such as cancer. In our study, we confirmed that SFTA1P was down-regulated in GC tissues compared with that in the adjacent normal tissues. SFTA1P could be a crucial biomarker of GC in clinical settings since the down-regulation of SFTA1P was related to poorer outcomes for patients with GC. Moreover, overexpression of SFTA1P could noticeably inhibit proliferation both *in vitro* and *in vivo*, promote G1 arrest, cause apoptosis of GC cells, and restrain GC cell migration and invasion. Interestingly, though SFTA1P similarly inhibits LUAD cell migration and invasion, it does not inhibit LUAD cell proliferation [23].

To date, the number of reports that have illuminated the participation of pseudogene-derived lncRNAs in human cancer development and progression remains limited. However, we know that lncRNAs may exert their effect on cell function through multiple ways. Studies show that pseudogene-derived lncRNAs can act as endogenous competitors for miRNAs and RNA-binding proteins (RBPs), and they may generate endogenous siRNA (esiRNA) and asRNA [28–31]. For example, Wang et al. demonstrated the existence of competition for miRNAs between the pseudogene transcript OCT4-pg4 and the functional gene transcript OCT4 in hepatocellular carcinoma (HCC). Both OCT4-pg4 and OCT4 target the tumor-suppressive miRNA *miR-145* directly. OCT4-pg4 functions as a natural miRNA sponge for *miR-145* and thereby protects the OCT4 transcript from being inhibited by *miR-145*, thus exerting an oncogenic role in HCC [32]. In addition, Chiefari et al. [33] showed that the transcript of the HMGA1 pseudogene (HMGA1-p) acts as a decoy for the trans-acting cytoplasmic protein αCP1. Elevated levels of HMGA1-p RNA compete for αCP1 thereby accelerating degradation of the *HMGA1* mRNA transcript [33]. Furthermore, Chan et al. [34] showed that the transcript of the pseudogene ψPPM1K could develop a tumor-suppressing ability independent of its parental gene. Moreover, Hawkins and Morris [35] found that OCT4 pseudogene 5 (OCT4-pg5) generates an asRNA that plays a negative role in the transcriptional regulation of OCT4.

A TP53 mutation is detected frequently in patients with GC and plays a critical role in tumor formation and progression [36]. A study showed that the deficiency of PICT1 could significantly inhibit cell proliferation by interfering with TP53-mediated cell cycle regulation of GC cells [37]. Moreover, Calcagno et al. [38] found that the copy number and mRNA expression of TP53 were lower in gastric tumors than in paired non-neoplastic specimens. In the present study, we hypothesized that TP53 was pertinent to the functions of SFTA1P in GC. In our study, increasing levels of *TP53* mRNA and protein were noted when SFTA1P was overexpressed, confirming our hypothesis. We propose that TP53 might be involved in the action of SFTA1P on cell proliferation, migration, and invasion. However, the concrete mechanistic connection between TP53 and SFTA1P in GC remains to be determined.

We confirmed for the first time that the pseudogene-derived lncRNA SFTA1P is down-regulated in GC specimens and its down-regulation may be linked with poorer outcomes for patients with GC. SFTA1P can inhibit cell proliferation, migration, and invasion possibly by interacting with TP53. Taken together, our results suggest that the pseudogene-derived lncRNA SFTA1P plays a tumor-suppressing role in the pathological process leading to GC. Our findings provide a new insight into the biological mechanism of GC and could lead to the development of a potential biomarker for the early diagnosis of GC, thereby improving its prognosis. Further exploration of pseudogenes and lncRNAs will deepen our understanding of the pathogenesis of this deadly disease.

## Abbreviations

asRNA: antisense RNA
ceRNA: competing endogenous RNA
GAPDH: glyceraldehyde-3-phosphate dehydrogenase
GC: gastric cancer
HCC: hepatocellular carcinoma
HMGA1-p: HMGA1 pseudogene
lncRNA: long non-coding RNA
LUAD: lung adenocarcinoma
PI: Propidium Iodide
qRT-PCR: Quantitative real-time reverse transcription PCR
SFTA1P: surfactant associated 1, pseudogene
